# Novel Intriguing Strategies Attenuating to Sarcopenia

**DOI:** 10.1155/2012/251217

**Published:** 2012-02-20

**Authors:** Kunihiro Sakuma, Akihiko Yamaguchi

**Affiliations:** ^1^Research Center for Physical Fitness, Sports and Health, Toyohashi University of Technology, 1-1 Hibarigaoka, Tenpaku-cho, Toyohashi 441-8580, Japan; ^2^School of Dentistry, Health Sciences University of Hokkaido, Kanazawa, Ishikari-Tobetsu, Hokkaido 061-0293, Japan

## Abstract

Sarcopenia, the age-related loss of skeletal muscle mass, is characterized by a deterioration of muscle quantity and quality leading to a gradual slowing of movement, a decline in strength and power, increased risk of fall-related injury, and, often, frailty. Since sarcopenia is largely attributed to various molecular mediators affecting fiber size, mitochondrial homeostasis, and apoptosis, the mechanisms responsible for these deleterious changes present numerous therapeutic targets for drug discovery. Resistance training combined with amino acid-containing supplements is often utilized to prevent age-related muscle wasting and weakness. In this review, we summarize more recent therapeutic strategies (myostatin or proteasome inhibition, supplementation with eicosapentaenoic acid (EPA) or ursolic acid, etc.) for counteracting sarcopenia. Myostatin inhibitor is the most advanced research with a Phase I/II trial in muscular dystrophy but does not try the possibility for attenuating sarcopenia. EPA and ursolic acid seem to be effective as therapeutic agents, because they attenuate the degenerative symptoms of muscular dystrophy and cachexic muscle. The activation of peroxisome proliferator-activated receptor **γ** coactivator 1**α** (PGC-1**α**) in skeletal muscle by exercise and/or unknown supplementation would be an intriguing approach to attenuating sarcopenia. In contrast, muscle loss with age may not be influenced positively by treatment with a proteasome inhibitor or antioxidant.

## 1. Introduction

Skeletal muscle contractions power human body movements and are essential for maintaining stability. Skeletal muscle tissue accounts for almost half of the human body mass and, in addition to its power-generating role, is a crucial factor in maintaining homeostasis. Given its central role in human mobility and metabolic function, any deterioration in the contractile, material, and metabolic properties of skeletal muscle has an extremely important effect on human health. Aging is associated with a progressive decline of muscle mass, quality, and strength, a condition known as sarcopenia [1]. The term sarcopenia, coined by I. H. Rosenberg, originates from the Greek words *sarx* (flesh) and *penia* (loss). Although this term is applied clinically to denote loss of muscle mass, it is often used to describe both a set of cellular processes (denervation, mitochondrial dysfunction, inflammatory and hormonal changes) and a set of outcomes such as decreased muscle strength, decreased mobility and function [2], increased fatigue, a greater risk of falls [3], and reduced energy needs [4]. In addition, reduced muscle mass in aged individuals has been associated with decreased survival rates following critical illness [5]. Estimates of the prevalence of sarcopenia range from 13% to 24% in adults over 60 years of age to more than 50% in persons aged 80 and older [2]. The estimated direct healthcare costs attributable to sarcopenia in the United States in 2000 were $18.5 billion ($10.8 billion in men and $7.7 billion in women), which represented about 1.5% of total healthcare expenditures for that year [6]. Therefore, age-related losses in skeletal muscle mass and function present an extremely important current and future public health issue. 

Lean muscle mass generally contributes up to ~50% of total body weight in young adults but declines with aging to be 25% at 75–80 yr old [7, 8]. The loss of muscle mass is typically offset by gains in fat mass. The loss of muscle mass is most notable in the lower limb muscle groups, with the cross-sectional area of the vastus lateralis being reduced by as much as 40% between the age of 20 and 80 yr [9]. On a muscle fiber level, sarcopenia is characterized by specific type II muscle fiber atrophy, fiber necrosis, and fiber-type grouping [9–13]. In elderly men, Verdijk et al. [12] showed a reduction in type II muscle fiber satellite cell content with aging. Although various investigators support such an age-related decrease in the number of satellite cells [12–17], some reports [18–20] indicate no such change. In contrast, most studies point to an age-dependent reduction in muscle-regenerative capacity due to reduced satellite cell proliferation and differentiation.

Several possible mechanisms for age-related muscle atrophy have been described; however, the precise contribution of each is unknown. Age-related muscle loss is a result of reductions in the size and number of muscle fibers [21] possibly due to a multifactorial process that involves physical activity, nutritional intake, oxidative stress, and hormonal changes [3, 22]. The specific contribution of each of these factors is unknown, but there is emerging evidence that the disruption of several positive regulators (Akt and serum response factor) of muscle hypertrophy with age is an important feature in the progression of sarcopenia [23–25]. In contrast, many investigators have failed to demonstrate an age-related enhancement in levels of common negative regulators [atrophy gene-1 (Atrogin-1), myostatin, and calpain] in senescent mammalian muscles [24, 25]. 

Resistance training combined with amino acid-containing supplements is effective candidate to prevent age-related muscle wasting and weakness [24–26]. In particular, sarcopenia has been most attenuated by treatment with many essential amino acids plus high-amount leucine [24–26]. In addition, many researchers have focused on inhibiting myostatin for treating various muscle disorders such as muscular dystrophy, cachexia, and sarcopenia [27, 28]. Furthermore, more recent studies have indicated a possible application of new supplements to prevent muscle atrophy [29, 30]. This review aims to address several novel strategies for inhibiting the muscle wasting in particular sarcopenia. 

## 2. Myostatin Inhibition

Growth and differentiation factor 8, otherwise known as myostatin, was first discovered during screening for members of a novel transforming growth factor-*β* (TGF-*β* ) superfamily and shown to act as a potent negative regulator of muscle growth [31, 32]. Studies indicate that myostatin inhibits cell-cycle progression and levels of myogenic regulatory factors, thereby controlling myoblastic proliferation and differentiation during developmental myogenesis [32–35]. Mutations in myostatin can lead to massive hypertrophy and/or hyperplasia in developing animals, as evidenced by knockout experiments in mice and by the phenotype seen in myostatin-null cattle [36] and humans [37]. Myostatin binds to and signals through a combination of Activin IIA/B receptors (ActRIIA/IIB) on the cell membrane; however, it has higher affinity for ActRIIB. On binding ActRIIB, myostatin forms a complex with a second surface type I receptor, either activin receptor-like kinase (ALK4 or ActRIB) or ALK5, to stimulate the phosphorylation of receptor Smad (Rsmad), and Smad2/3 transcription factors in the cytoplasm. Then Smad2/3 translocate and modulate nuclear gene transcription such as MyoD [33] via a TGF-*β*-like mechanism. In contrast, forkhead box O (FOXO) 1 and Smad2 appear to control the differentiation of C2C12 myoblasts by regulating myostatin mRNA and its promoters [38]. 

Studies measuring myostatin levels during aging have yielded conflicting results such as marked increases in humans at the mRNA and protein levels [39], no change in mice at the protein level [40], and a decrease in rats at the mRNA level [41]. The functional role of myostatin in aged mammalian muscle may be revealed by further descriptive analysis using other methods (ex. immunofluorescence) and examining the adaptive changes in downstream modulators (ex. ActRIIB, Smad3) of myostatin signaling.

Many researchers have focused on inhibiting myostatin for treating various muscle disorders. The use of neutralizing antibodies to myostatin improved muscle disorders in rodent models of Duchenne muscular dystrophy (DMD), limb girdle muscular dystrophy 2F (Sgcg^−/−^), and amyotrophic lateral sclerosis (SOD1^G93A^ transgenic mouse) [27, 28, 42, 43]. Indeed, myostatin inhibition using MYO-029 was tested in a prospective, randomized, and US phase I/II trial in 116 adults with muscular dystrophy such as Becker muscular dystrophy, facioscapulohumeral muscular dystrophy, and limb-girdle muscular dystrophy [44]. On the other hand, inhibiting myostatin to counteract sarcopenia has also been investigated only in animals. A lack of myostatin caused by gene manipulation increased the number of satellite cells and enlarged the cross-sectional area of predominant type IIB/X fibers in tibialis anterior muscles of mice [45]. In addition, these myostatin-null mice showed prominent regenerative potential including accelerated fiber remodeling after an injection of notexin [45]. LeBrasseur et al. [46] reported several positive effects of 4 weeks of treatment with PF-354 (24 mg/Kg), a drug for myostatin inhibition, in aged mice. They showed that PF-354-treated mice exhibited significantly greater muscle mass (by 12%), and increased performance such as treadmill time, distance to exhaustion, and habitual activity. Furthermore, PF-354-treated mice exhibited decreased levels of phosphorylated Smad3 and muscle ring-finger protein 1 (MuRF1) in aged muscle. More recently, Murphy et al. [47] showed, by way of once weekly injections, that a lower dose of PF-354 (10 mg/Kg) significantly increased the fiber cross-sectional area (by 12%) and in situ force of tibialis anterior muscles (by 35%) of aged mice (21-mo-old). In addition, this form of treatment reduced markers of apoptosis by 56% and reduced caspase3 mRNA levels by 65%. Blocking myostatin enhances muscle protein synthesis [48] by potentially relieving the inhibition normally imposed on the Akt/mammalian target of rapamycin- (mTOR) signaling pathway by myostatin [49]. The blockade may also attenuate muscle protein degradation by inhibiting the ubiquitin-proteasome system, which is controlled, in part, by Akt [50, 51] although the mechanism involved has not been demonstrated. In contrast, a microarray analysis of the skeletal muscle of myostatin knockout mice showed an increased expression of antiapoptotic genes compared with that in control mice [51]. These lines of evidence clearly highlight the therapeutic potential of antibody-directed inhibition of myostatin for treating sarcopenia by inhibiting protein degradation and/or apoptosis.

## 3. Ursolic Acid

A water-insoluble pentacyclic triterpenoid, ursolic acid is the major waxy component in apple peels [52]. It is also found in many other edible plants. Interestingly, because it exerts beneficial effects in animal models of diabetes and hyperlipidemia [53, 54], ursolic acid is thought to be the active component in a variety of folkloric antidiabetic herbal medicines [53, 55]. As predicted by connectivity mapping, Kunkel et al. [29] found that ursolic acid reduced skeletal muscle atrophy in the setting of two-distinct atrophy-inducing stresses (fasting and muscle denervation). A major strength of the connectivity map is that it takes into account positive and negative changes in mRNA expression that together constitute an authentic mRNA expression signature. Thus, by querying the connective map with signatures of muscle atrophy, Kunkel et al. [29] were, in effect, querying with the reciprocal signature of muscle atrophy but also induced muscle hypertrophy. 

Ursolic acid might increase muscle mass by inhibiting atrophy-associated skeletal muscle gene expression. Indeed, Kunkel et al. [29] found that acute ursolic acid treatment of fasted mice reduced Atrogin-1 and MuRF1 mRNA levels in association with reduced muscle atrophy. Similarly, chronic ursolic acid treatment of unstressed mice reduced Atrogin-1 and MuRF1 expression and induced muscle hypertrophy. Although ursolic acid increased skeletal muscle Akt phosphorylation *in vivo*, the experiments could not determine if it acted directly on skeletal muscle, how quickly it acted, and if the effect required insulin-like growth factor-I (IGF-I) or insulin, which are always present in healthy animals, even during fasting. To address these issues, Kunkel et al. [29] studied serum-starved skeletal myotubes and found that ursolic acid rapidly stimulated IGF-I receptor and insulin receptor activity, but only if IGF-I or insulin was also present. Taken together, their data suggest that ursolic acid first enhances the capacity of preexisting IGF-I and insulin to activate skeletal muscle IGF-I receptors and insulin receptors, respectively. Importantly, ursolic acid alone was not sufficient to increase phosphorylation of the IGF-I receptor or insulin receptor. Rather, its effects also required IGF-I and insulin, respectively. This suggests that ursolic acid either facilitates hormone-mediated receptor autophosphorylation or inhibits receptor dephosphorylation. The latter possibility is supported by previous *in vitro* data showing that ursolic acid directly inhibits PTP1B [56], a tyrosine phosphatase that dephosphorylates (inactivates) the IGF-I and insulin receptors [57]. Further research is needed to elucidate the effect of supplementation with ursolic acid in skeletal muscle and to attenuate muscle wasting (ex. sarcopenia).

## 4. Eicosapentaenoic Acid

Eicosapentaenoic acid (EPA) is a 20-carbon omega (n)-3 polyunsaturated fatty acid with anti-inflammatory properties, which is synthesized from ingested alpha-linolenic acid or is consumed in fish and fish oil such as cod liver, sardine, and salmon oil [58]. There is no established Dietary Reference Intake for n-3 fatty acids; yet, adequate intake (AI) is set at 1.6 and 1.1 g/d for men and women, respectively. While intake in the United States occurs at levels much lower than the proposed AI and no signs of deficiency are observed, the AI is proposed to provide optimal health benefits associated with consuming omega-3 polyunsaturated fatty acids [59]. Several clinical trials have reported potential health benefits of omega-3 polyunsaturated fatty acids in many diseases, including cardiovascular diseases [60], epilepsy, inflammatory bowel disease, exercise-trained subjects [61], and cancer-associated cachexia [62]. In particular, the administration of omega-3 fatty acids and EPA capsules or supplements with EPA has been shown to be associated with weight stabilization, gains in lean body mass, and improvements in quality of life markers in weight-losing patients with advanced pancreatic cancer. In addition, EPA has also been shown to inhibit the proinflammatory transcription factor nuclear factor kappaB (NF-*κ*B) [62, 63], to reduce tumor necrosis factor-*α* (TNF-*α*) production by macrophages [64] and to prevent the damaging effects of TNF-*α* during skeletal muscle differentiation *in vitro* [65]. Furthermore, short-term treatment with EPA (16 day, 100 mg/Kg) attenuates the muscle degeneration of mdx mice, a model of DMD [66]. EPA treatment decreased creatine kinase levels and attenuated myonecrosis (decrease in Evans-blue dye-positive fibers and a concomitant increase in peripheral nucleated fibers), and reduced the levels of TNF-*α*. 

Some evidence suggests omega-3 polyunsaturated fatty acids to be also a potentially useful therapeutic agent for the treatment and prevention of sarcopenia. In a more recent study [30], sixteen healthy, older adults have been randomly assigned to receive either omega-3 fatty acids or corn oil for 8 week. In their study, the rate of muscle protein synthesis and the phosphorylation of key elements of the anabolic-signaling pathway were evaluated in three different conditions. Smith et al. [30] found that omega-3 fatty acid supplementation had no effect on the basal rate of muscle protein synthesis but augmented the hyperaminoacidemia- hyperinsulinemia-induced increase in the rate of muscle protein synthesis probably due to a greater increase in muscle p70S6K^ Thr389^ phosphorylation.

## 5. Angiotensin-Converting Enzyme Inhibitors

Angiotensin-converting enzyme (ACE) inhibitors have long been used as a treatment in primary and secondary prevention in cardiovascular disease as well as secondary stroke prevention. It has now been suggested that ACE inhibitors may have a beneficial effect on skeletal muscle. ACE inhibitors may exert their beneficial effects on skeletal muscles through a number of different mechanisms. ACE inhibitors may improve muscle function through improvements in endothelial function, metabolic function, anti-inflammatory effects, and angiogenesis thereby improving skeletal muscle blood flow. ACE inhibitors can increase mitochondrial numbers and IGF-I levels thereby helping to counter sarcopenia [67–69]. 

Observational studies have shown that the long-term use of ACE inhibitors was associated with a lower decline in muscle strength and walking speed in older hypertensive people and a greater lower limb lean muscle mass when compared with users of other antihypertensive agents [70]. Several studies have shown that ACE inhibitors improved exercise capacity in both younger and older people with heart failure [70, 71] but caused no improvement in grip strength [72]. Although this could be largely attributed to improvements in cardiac function, skeletal muscle atrophy is also associated with chronic heart failure so the evidence of muscle gains should not be discounted. Few interventional studies using ACE inhibitors for physical function have been undertaken. One study looking at functionally impaired older people without heart failure has shown that ACE inhibitors increase 6-minute walking distance to a degree comparable to that achieved after 6 months of exercise training [73]. Another found that ACE inhibitors increased exercise time in older hypertensive men [74]. However, a study comparing the effects of nifedipine with ACE inhibitors in older people found no difference between treatments in muscle strength, walking distance, or functional performance [75]. It is possible that frailer subjects with slower walking speeds, who have a tendency to more cardiovascular problems, benefit more. Further evidence is required before recommending ACE inhibitors to counter the effects of sarcopenia. However, ACE inhibitors are associated with cardiovascular benefits, and as older people frequently have underlying cardiovascular problems, these agents are already commonly prescribed.

## 6. Proteasome Inhibitors

In a variety of conditions such as cancer, diabetes, denervation, uremia, sepsis, disuse, and fasting, skeletal muscles undergo atrophy through degradation of myofibrillar proteins via the ubiquitin-proteasome pathway [76]. Recent advances have asserted that muscle atrophy in these conditions shares a common mechanism in the induction of the muscle-specific E3 ubiquitin ligases Atrogin-1 and MuRF1 [77–79]. Only very indirect measurements (small increases in mRNA levels encoding some components of the ubiquitin-proteasome pathway [80–82] or ubiquitin-conjugate accumulation [83]) in old muscles of rodents or humans suggested a modest activation of this pathway. Atrogin-1 and/or MuRF1 mRNA levels in aged muscle are reportedly unchanged in humans [83, 84], increased in rats [80, 85], or decreased in rats [82, 86, 87]. When even the mRNA expression of these atrogenes increased in sarcopenic muscles, the induction was very limited (1.5–2.5-fold) as compared with other catabolic situations (10-fold). In addition, the major peptidase activities of the proteasome (i.e., the chymotrypsin-like, trypsin-like, and caspase-like activities) were always reduced (as reported in other tissues [88]) or unchanged with aging [78, 81, 88, 89]. Altogether, these observations clearly suggest that activation of the ubiquitin-proteasome system contributed little to the establishment of sarcopenia in accordance with the very slow muscle mass erosion.

 There are several chemical classes of compounds that inhibit proteasomal activity, including peptide analogs of substrates with different C-terminal groups, such as aldehydes, epoxyketones, boronic acids, and vinyl sulfones [90]. A selective boronic acid proteasome inhibitor, Velcade (also known as PS-341 and bortezomib), directly inhibits the proteasome complex without direct effects on ubiquitination. Velcade is well distributed in the body and does not cross the blood-brain barrier. In addition to being useful research tools for dissecting the roles of the proteasome, proteasome inhibitors have potential applications in biotechnology and medicine. For example, through their ability to block the activation of NF-*κ*B, proteasome inhibitors can dramatically reduce *in vitro* and *in vivo* the production of inflammatory mediators as well as of various leukocyte adhesion molecules, which play a crucial role in many diseases. Indeed, Velcade is orally active and is presently approved by the Food and Drug Administration and the European Medicines Agency and well tolerated for treating multiple myeloma [91, 92]. Bonuccelli et al. [93] had indicated that Velcade, once injected locally into the gastrocnemius muscles of mdx mice, could upregulate the expression and membrane localization of dystrophin and members of the dystrophin-glycoprotein complex. Gazzerro et al. [94] suggested that treatment with Velcade (0.8 mg/Kg) over a 2-week period reduced muscle degeneration and necrotic features in mdx muscle fibers, as evaluated with Evans blue dye. In addition, they observed many myotubes and/or immature myofibers expressing embryonic myosin heavy chain in mdx muscle after Velcade administration probably due to the upregulation of several myogenic differentiating modulators (MyoD and Myf-5). These effects of Velcade on muscle degeneration would differ dependent on muscle-fiber type. Beehler et al. [95] demonstrated selective attenuation on treatment with Velcade (3 mg/Kg, 7 days) for atrophy of denervated slow-type muscle (soleus), but not fast-type muscle (EDL) of rats. In contrast, MG-132 exerts an inhibitory effect on both the proteasome and the calpain system. More recently, Gazzerro et al. [94] have clearly demonstrated that MG-132 increased dystrophin, alpha-sarcoglycan, and beta-dystroglycan protein levels in explants from Becker muscular dystrophy patients, whereas it increased the proteins of the dystrophin glycoprotein complex in DMD cases. Strangely, there is no rodent study examining the effect of these proteasome inhibitors to prevent muscle atrophy with aging. As indicated previously [80, 82, 88, 89], almost no studies demonstrated an enhancement of proteasome-linked modulators for protein degradation in sarcopenic mammalian muscles. Proteasome inhibitors may not act to attenuate muscle wasting in cases of sarcopenia.

## 7. Cyclophilin Inhibitor (Debio-025)

Ca^2+^ overload is known to cause cellular necrosis by directly inducing the opening of the mitochondrial permeability transition (MPT) pore [96, 97]. The MPT pore spans the inner and outer membranes of the mitochondria and, when opened for prolonged periods of time, leads to loss of ATP generation, swelling, rupture, and induction of cell death [96, 97]. Cyclophilin D is a mitochondrial matrix prolyl cis-trans isomerase that directly regulates calcium—and reactive oxygen species-dependent MPT and cellular necrosis. Indeed, mice lacking Ppif (the gene-encoding cyclophilin D) show protection from necrotic cell death in the brain and heart after ischemic injury, and mitochondria isolated from these mice are resistant to calcium-induced swelling [98, 99]. Additionally, genetic deletion of Ppif attenuated various dystrophic symptoms (fiber atrophy, fiber loss, invasion by inflammatory cells, and swollen mitochondria) of mice lacking *δ*-sarcoglycan and the **α**2-chain of laminin-2 [100]. Millay et al. [100] demonstrated that the subcutaneous injection of Debio-025, a potent inhibitor of the cyclophilin family, improved calcium overload-induced swelling of mitochondria and reduced manifestations of necrotic disease such as fibrosis and central nuclei, in mdx mice, a model of DMD. In addition, treatment with Debio-025 prevented mitochondrial dysfunction and normalized the apoptotic rates and ultrastructural lesions of myopathic Col6a1^−/−^ mice, a model of human Ullrich congenital muscular dystrophy and Bethlem myopathy [101]. More recently, orally administered Debio-025 reduced creatine kinase blood levels and improved grip strength in mdx mice after 6 weeks of treatment [102]. This effect on muscular dystrophy was greater than that of prednisone, currently the standard for treatment of DMD [103, 104]. However, it had not been examined until now whether Debio-025 also has a therapeutic effect on the loss and/or atrophy of muscle fiber with aging in rodents as well as humans. Since there are many symptoms in common between muscular dystrophy and sarcopenia, treatment with Debio-025 may counteract sarcopenic symptoms.

## 8. PGC-1**α**

Although the mechanisms by which calorie restriction (CR, 30–40%) delays the aging process remain to be fully elucidated, CR is intricately involved in regulating cellular and systemic redox status and in modulating the expression of genes related to macromolecule and organelle turnover, energy metabolism, and cell death and survival [109–111]. Several studies indicate the protection of age-related functional decline and loss of muscle fibers by CR [109, 110]. These protective effects are likely attributable to the ability of CR to reduce the incidence of mitochondrial abnormalities (mitochondrial proton leak), attenuate oxidative stress [reactive oxidative species (ROS) generation], and counteract the age-related increases in proapoptotic signaling in skeletal muscle [109, 112]. Therefore, several lines of evidence suggest mitochondrial involvement in sarcopenia. Therapeutic strategies for sarcopenia like endurance exercise [113] and CR [114] result in increased mitochondrial capacity in the muscle. A key player controlling mitochondrial function is the peroxisome proliferator-activated receptor *γ* coactivator *α* (PGC-1*α*), a master regulator of mitochondrial biogenesis. In skeletal muscle, PGC-1*α* can also prevent muscle wasting by regulating autophagy [115] and stabilization of the neuromuscular junction program [116] in the context of muscle atrophy during disease. Thereby, PGC-1*α* links mitochondrial function to muscle integrity [115]. PGC-1*α* levels in skeletal muscle decrease during aging [116]. The health-promoting effects of increased PGC-1*α* expression in skeletal muscle have been shown in different mouse models with affected muscle such as DMD [117], denervation-induced atrophy [115], and mitochondrial myopathy [118]. Indeed, Wenz et al. [118] showed that elevated PGC-1*α* expression in skeletal muscle enhanced oxidative phosphorylation function in a mouse model of mitochondrial myopathy, delaying the onset of the myopathy and markedly prolonging lifespan. 

**Figure 1:**
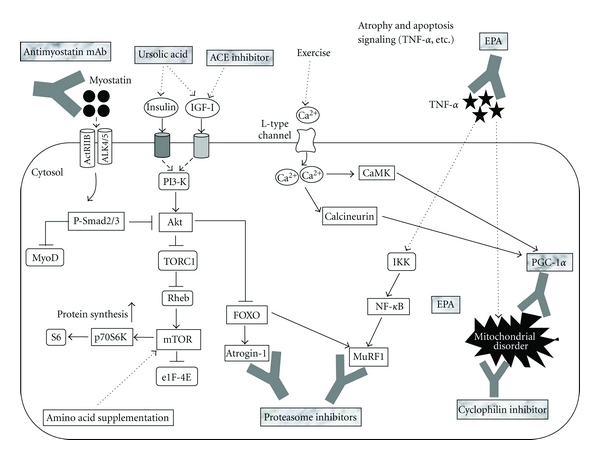
Myostatin signals through the ActRIIB-ALK4/5 heterodimer activate Smad2/3 with blocking of MyoD transactivation in an autoregulatory feedback loop. In addition, Smad3 sequesters MyoD in the cytoplasm to prevent it from entering the nucleus and activating the stem cell population. Recent findings [105, 106] have suggested that myostatin-Smad pathway inhibits protein synthesis probably due to blocking the functional role of Akt. Supplementation with ursolic acid upregulates the amount of IGF-I and insulin and then stimulates protein synthesis by activating Akt/mTOR/p70S6K pathway [29]. Treatment with ACE inhibitor also enhances IGF-I level in muscle. Amino acid supplementation enhances protein synthesis by stimulating mTOR [107]. Akt blocks the nuclear translocation of FOXO to inhibit the expression of Atrogin-1 and MuRF1 and the consequent protein degradation. Proteasome inhibitors combat the ubiquitin-proteasome signaling activated by these atrogenes. In cachexic muscle, supplementation with EPA downregulates the amount of TNF-*α* and NF-*κ*B [63, 64]. Endurance exercise increases the amount of PGC-1*α* through calcineurin- or CaMK-dependent signaling [108]. Both activated PGC-1*α*, and cyclophilin inhibitor protects several mitochondrial disorders (apoptosis, oxidative damage, etc.) elicited by the increase in NF-*κ*B and Bax and/or the decrease in Bcl-2 in senescent muscle. ACE; angiotensin-converting enzyme, ActRIIB; activin receptor IIB, ALK4/5; activin-like kinase 4/5, CaMK; calmodulin kinase, eIF4E; eukaryotic initiation factor 4E, EPA; eicosapentaenoic acid, FOXO; Forkhead box O, IGF-I; insulin-like growth factor-I, IKK; inhibitor of *κ*B kinase, mTOR; mammalian target of rapamycin, MuRF1; muscle ring-finger protein 1, NF-*κ*B; nuclear factor of kappa B, PGC-1*α*; peroxisome proliferator-activated receptor *γ* coactivator *α*, PI3-K; phosphatidylinositol 3-kinase, Rheb; Ras homolog enriched in brain, TNF-*α*; tumor nectosis factor-*α*, TORC1; a component of TOR-signaling complex 1.

Adipose tissue infiltration of skeletal muscle also increases with age [119, 120]. Sarcopenia may be linked with increased obesity in the elderly [121]. Indeed, persons who are obese and sarcopenic are reported, independent of age, ethnicity, smoking, and comorbidity, to have worse outcomes, including functional impairment, disabilities, and falls, than do those who are nonobese and sarcopenic [122]. Recent work has demonstrated that mitochondrial damage occurs in obese individuals due to enhanced ROS and chronic inflammation caused by increased fatty acid load [123–125]. Specifically, in skeletal muscle, the expression of PGC-1*α* drives not only mitochondrial biogenesis and the establishment of oxidative myofibers, but also vascularization [126, 127]. It was found that a high-fat diet or fatty acid treatment caused a reduction in the expression of PGC-1*α* and other mitochondrial genes in skeletal muscle [128], which may be a mechanism through which excess caloric intake impairs skeletal muscle function. A recent study has also demonstrated that transgenic overexpression of PGC-1*α* in skeletal muscle improved sarcopenia and obesity associated with aging in mice [129]. Endurance training has been shown to upregulate the amount of PGC-1*α* to elicit mitochondrial biogenesis [130]. The well-known sarcopenia-attenuating effects by endurance training may be attributable to the protection for mitochondrial disorders (apoptosis, oxidative damage, etc.) by the increase of PGC-1*α* amount.  Figure 1 provides an overview of the molecular pathways of muscle hypertrophy and novel strategies for counteracting sarcopenia.

## 9. Conclusions and Perspectives

The advances in our understanding of muscle biology that have occurred over the past decade have led to new hopes for pharmacological treatment of muscle wasting. These treatments will be tested in humans in the coming years and offer the possibility of treating sarcopenia/frailty. These treatments should be developed in the setting of appropriate dietary and exercise strategies. Currently, resistance training combined with amino acid-containing supplements would be the best way to prevent age-related muscle wasting and weakness. Supplementation with ursolic acid and EPA seems to be more intriguing candidates combating sarcopenia although systematic and fundamental research in these treatments has not been conducted even in rodent. The well-known sarcopenia-attenuating effects by endurance training may be attributable to the protection for mitochondrial disorders by the increase of PGC-1*α* amount. 

## Abbreviations

ACE: Angiotensin-converting enzyme
ActRIIB: Activin receptor IIB
AI: Adequate intake
ALK: Activin receptor-like kinase
Atrogin-1: Atrophy gene-1
CR: Calorie restriction
DMD: Duchenne muscular dystrophy
EPA: Eicosapentaenoic acid
FOXO: Forkhead box O
IGF-I: Insulin-like growth factor-I
MPT: Mitochondrial permeability transition
mTOR: Mammalian target of rapamycin
NF-*κ*B: Nuclear factor-kappaB
PGC-1*α*: Peroxisome proliferator-activated receptor *γ* coactivator 1*α*
ROS: Reactive oxidative species
TGF-*β*: Transforming growth factor-*β*
TNF-*α*: Tumor necrosis factor-*α*.
